# A rule-free workflow for the automated generation of databases from scientific literature

**DOI:** 10.1038/s41524-023-01171-9

**Published:** 2023-12-13

**Authors:** Luke P. J. Gilligan, Matteo Cobelli, Valentin Taufour, Stefano Sanvito

**Affiliations:** 1 School of Physics, AMBER and CRANN Institute, Trinity College, Dublin 2, Dublin, Ireland; 2 Department of Physics and Astronomy, University of California, Davis, CA 95616 USA

**Keywords:** Computational methods, Electronic structure

## Abstract

In recent times, transformer networks have achieved state-of-the-art performance in a wide range of natural language processing tasks. Here we present a workflow based on the fine-tuning of BERT models for different downstream tasks, which results in the automated extraction of structured information from unstructured natural language in scientific literature. Contrary to existing methods for the automated extraction of structured compound-property relations from similar sources, our workflow does not rely on the definition of intricate grammar rules. Hence, it can be adapted to a new task without requiring extensive implementation efforts and knowledge. We test our data-extraction workflow by automatically generating a database for Curie temperatures and one for band gaps. These are then compared with manually curated datasets and with those obtained with a state-of-the-art rule-based method. Furthermore, in order to showcase the practical utility of the automatically extracted data in a material-design workflow, we employ them to construct machine-learning models to predict Curie temperatures and band gaps. In general, we find that, although more noisy, automatically extracted datasets can grow fast in volume and that such volume partially compensates for the inaccuracy in downstream tasks.

## Introduction

Since the dawn of modern science, there has been a continuous, exponential growth in the volume of published scientific literature^1^. When related to materials science, such an abundance of data clearly offers a wide range of possibilities and opportunities. Materials data, in fact, can provide the foundation for models and theories to navigate the physical/chemical space and ultimately drive discovery. Unfortunately, access to information from unstructured literature at such a massive scale presents significant technical and practical challenges. As a result, in general, curated databases are scarce and often limited to theoretical data only. This is because for theory data, one does not need to exploit the literature, but simply run highly standardized first-principles calculations, which are amenable to automated collection^2–5^. Importantly, large-scale theoretical datasets have been proven to be a revolutionary tool in the search for new materials with unique properties and for the discovery of intricate materials trends. For instance, they have been used to predict the existence of novel magnets^6^, to identify materials regions favorable to superconductivity^7^, to design novel high-entropy alloys^8^, to identify low-thermal-conductivity compounds^9^, or to predict the *zT* thermoelectric figure of merit in inorganic materials^10^, just to name a few examples. Furthermore, theory datasets have been a platform for constructing machine learning (ML) models with enhanced throughput^11–13^.

Although no calculation can replace an experiment, databases containing experimental results are much rarer and typically smaller. In general, these are extremely labor-intensive to generate, since the information is not directly collated at the laboratories but needs to be mined from the published literature. As a consequence, experimental databases are not comprehensive but usually list only a handful of properties for each compound, such as the crystal^14–16^ or magnetic structure^17^. Furthermore, they update slowly and, given the effort needed to construct them, they are often proprietary in nature, despite the efforts of some open-access initiatives^14,17^. Thus, the existing landscape of experimental datasets is incomplete and fragmented, and most of the known experimental results remain accessible only through unstructured scientific literature.

This is a serious drawback for materials innovation, not only because experimental data corresponds to reality but also because experimental data may often contain information inaccessible to simulations or may be located where simulations are prohibitive or highly inaccurate. It is also important to note that typical ML models for property predictions are often very data-hungry, so the absence of large datasets of experimental data precludes their formulation. Still, there have been a few successful examples of the use of experimental data only to construct ML predictors and classifiers for materials properties^18–20^ and examples of transfer-learning between theoretical and experimental data^21^. These suggest that the creation of structured experimental databases may represent a significant asset in materials design.

Given the large volume of literature regularly published, this appears to be a daunting task. In order to understand the scale of the problem, consider that there are 231 journals listed in the ‘materials science and engineering’ category of the Clarivate Master Journal List (mjl.clarivate.com/home). If the average number of articles published yearly in a journal is ~1000, we will have about a quarter of a million articles with material information published per year. Clearly, experimental databases of such capacity cannot be curated with laborious manual methods, indicating that automatization is the only way possible. The current state-of-the-art method for automatizing the data-extraction process from literature is ChemDataExtractor^22^. ChemDataExtractor relies upon rules-based text parsing coupled with conditional random fields for named entity recognition (NER)^23^. Hence, the performance of a new model depends on the ability of the user to adequately define rules. Furthermore, since different quantities can be described by natural language in structurally different ways, every new extraction task requires the user to define new grammatical and syntactic rules. These features make the process still labor-intensive and reduce the large-scale deployment of such methods.

In recent years, significant progress has been made in constructing tools that improve our ability to automate further this extraction procedure. Textual representations that are suitable for advanced, context-aware, natural language processing (NLP) have long been a focus of research in this domain. Simple representations such as one-hot encoding of dictionaries of vocabulary, bag of words models, or statistical representations weighting the importance of certain terms over others, such as term frequency-inverse document frequency (TF-IDF)^24^, have been the go-to representations for many of the more conventional NLP tasks. For instance, they have been successfully used in sentiment analysis or simple classification tasks. These representations are still adequate for handling large-scale texts with simple models. However, in order to operate on a level at which the extracted individual terms or sentences are processed accurately, we must turn to more sophisticated methods of representation.

Word embedding naturally follows as a viable candidate for such applications. These are textual representations that aim to describe words in a vocabulary as vectors in a high-dimensional vector space. In this space, the similarity between words is captured by the projection of one such vector onto the other. There are numerous algorithms to learn embeddings from a corpus of documents. Examples of the most widely used algorithms are word2vec^25^ and GloVe^26^, models that have also been used for scientific text embedding. For instance, the word2vec algorithm was used by the Materials Genome Initiative to train a domain-specific materials science embedded representation^27^. This embedding was demonstrated to exhibit a good knowledge of the chemical space, and insights into the physical properties of materials could even be extracted from purely text-based representations trained on scientific literature alone.

Transformer networks elaborate on these ideas and are the current state of the art in the representation of natural language^28^. The core concept of a transformer network is the use of self-attention to capture the syntactic interdependencies between words, meaning that these networks exhibit a superior ability to parse the context in which a term appears in a sentence. Arguably, one of the most prevalent transformer-based models for NLP is the Bidirectional Encoder Representations from Transformers (BERT), a large-scale architecture with several hundred million tuneable parameters^29^. Since its conception in 2018, BERT has rapidly become the language platform for models in many NLP applications, achieving state-of-the-art performance across a range of benchmarks^30,31^. Furthermore, there have been a number of BERT architectures adapted to outperform the conventional BERT model in various disparate NLP domains, including but not limited to the fields of general scientific literature^32^, financial sentiment analysis^33^, and biomedical text-mining^34^. There is also a previously fine-tuned architecture for the field of materials science, called MatSciBERT^35^, which is one of the pre-trained architectures we have employed in this work.

Currently, much focus is being placed on the use of autoregressive large language models, as a means of achieving state-of-the-art performance in a large variety of natural language tasks. While these models are giving promising results, there are some significant drawbacks associated with their use. Firstly, their size imposes heavy hardware requirements at inference time and even larger requirements for fine-tuning. It is possible to use services provided by private companies, such as OpenAI, to have access to these models through API. However, the fees can become prohibitive for the large number of prompts needed by an information-extraction workflow. It is also worth considering that for such large generative language models, there is little to no ecosystem of domain-specific pre-training, a feature of the BERT architectures. Indeed, non-domain-specific generative models have not been shown to be superior to domain-specific BERTs in the field of material science. Finally, generative models are known to “hallucinate", meaning that they currently have a tendency to make up false information in the generated text. When processing large amounts of data, it is impossible to verify that a generative model has not manufactured some of the data in the resulting database. This is a particularly critical problem in the context of the automatic generation of databases, since each entry is required to be reliably linked to its source. Common strategies to limit the effects of hallucinations include iteratively prompting to double-check the output. However, these approaches rapidly increase the number of tokens passed as prompt, hence the computation time and/or the API access cost for each extraction. For these reasons, we believe that an information-extraction workflow based on domain-specific BERT can be useful for the community, since it can be deployed and fine-tuned on commonly available hardware.

In this work, we introduce a rule-free workflow for the automatic extraction of information from scientific literature. The extraction is performed by mean of a sequence of BERT models finely tuned on specific downstream tasks. The superior contextual awareness of the BERT representation allows the necessary grammatical and syntactic rules for extraction to be learnt by the transformer model from a sample of labeled text. Thus, the text labeling step now substitutes the design of a rule-based grammar and it does not require any previous knowledge of natural language processing or coding to develop new extraction procedures. This is enabled by our self-contained literature-to-structured-properties database pipeline, which is here named BERT Precise Scientific Information Extractor (BERT-PSIE). Moreover, by leveraging the transfer-learning capabilities of BERT models, the convergence in performance on the downstream tasks is reached with a relatively small training set of labeled text. Each entry of the database generated by BERT-PSIE can be linked to a specific source without any possibility of hallucination, since all the language models used are trained for classification tasks and not used in a generative setting. Note that the work presented here is capable of extracting data only from text, but not from tables or images. An expansion of our capabilities to forms of information dissemination different from text is highly desirable and will be subject of future work.

Value has been already demonstrated for databases automatically generated with previously developed schemes, providing useful insights into a range of physical and chemical properties of compounds^36–38^. More recently, there have also been several inroads made in the development of transformers for materials science applications^39,40^. However, all these attempts present a common shortcoming. Namely, when validating the quality of the dataset automatically generated by NLP methods, one does not have available a reference set of manually curated entries to compare with. This means that the automatically generated datasets cannot be tested against an established ground truth reference. This issue is addressed here by choosing the properties for which we can obtain a manually curated dataset. The first of these properties is the Curie temperature, *T*_C_, for which we avail of the combined manually curated databases from Nelson et al.^18^ and Byland et al.^41^, and of one automatically generated using ChemDataExtractor^42^. The second such property is the electronic band gap, for which has a manually curated dataset aggregated by Zhuo et al.^20^. We will show that with a modest amount of labeled data, it is possible to train models that have performance on par with the state-of-the-art rule-based methods. Furthermore, we demonstrate that the automatically generated data can be used to construct both *T*_C_ and band gap predictors, such that they can already be used for predicting material properties.

We believe that the extensive and rigorous set of benchmarks, introduced in this work should be taken as a standard in the proposal of novel extraction models. This will enable a thorough comparison highlighting strengths and weaknesses, a comparison that appears increasingly necessary as the deployment of large language models in materials science becomes more widespread^43,44^.

## Results

### BERT-PSIE Structure

In this section, we discuss the performance of the NLP workflow implemented in BERT-PSIE for the automated extraction of structured data from scientific literature. BERT-PSIE is based on the concatenation of different BERT models, fine-tuned to perform the specific tasks necessary for the text-mining pipeline. Our designed workflow can be adapted to the extraction of any binary-related information and can potentially be extended to more complex form relations. In this work, we focus on mining the *T*_C_ of ferromagnets and the electronic band gap of semiconductors/insulators because of the availability of manually curated databases, which can be used as ground truth in assessing performance.

It is certainly true that the extraction of inter-related properties (for instance, a materials property may depend on the experimental temperature) can be more complex, but these are difficult to benchmark because of the lack of manually curated datasets. In such case, the extraction should provide the compound and all the inter-related quantities, simultaneously. The fact that BERT-PSIE works at the sentence-level may pose a limit to this, since the different information may be contained in different sentences. Strategies to overcome such limitations certainly deserve future work.

**Fig. 1:**
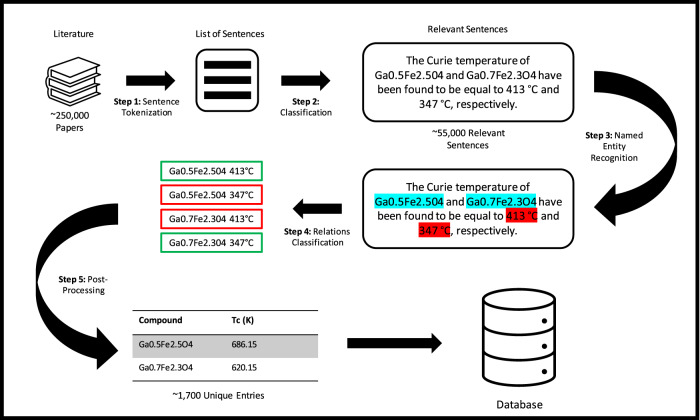
Schematic diagram of the BERT-PSIE pipeline for the automated extraction of compound-property pairs from the scientific literature. The workflow relies on the combination of BERT models fine-tuned for different downstream tasks such as sentence classification, named entity recognition, and relation classification. Here we use the Curie temperature as an example. See text for more details.

The structure of our entire workflow can be appreciated by looking at the scheme in Fig. 1. Papers are downloaded from the web by using a keyword-based search with the Crossref REST API (api.crossref.org). A classifier identifies the relevant sentences from the downloaded corpus, and a Named Entity Recognition (NER) module extracts material-property relations from sentences containing single unambiguous relations. Note that sentences containing a single entity only, either the compound or the property, are discarded. Then, a second module performs relation classification for sentences where multiple mentions of compounds and/or material’s properties are present. The material-property relations extracted then form the database.

Results are now presented for the entire workflow. In each of the following sections, we will first consider the database of Curie temperatures extracted with BERT-PSIE and then the database of electronic band gaps. Firstly, we discuss the performance (precision, recall, and *F*_1_ score) achieved by the three main modules. Then, we compare the automatically extracted database with our manually curated ones^18,41,20^ and those created with ChemDataExtractor^42^. Finally, we will evaluate the performance of this database in the screening for magnetic compounds by training ML models to predict the property in question.

### BERT models fine-tuning

Let us discuss the Curie temperature database first. The evaluation metrics for the sentence-level relevancy classifier are presented in the upper row of Table 1. Precision, *P*, and recall, *R*, are both above 0.8, indicating high-level model performance on the test data. Care was taken to ensure that the training data was as representative as possible of the literature. However, similarities in the syntactic structure reporting a temperature value are unavoidable, a fact that increases the level of noise in the extracted data. For example, consider the sentence ‘Barium titanate (BaTiO_3_) is a ferroelectric with a Curie temperature of 120 ^∘^C’. In this case, ‘Curie temperature’ refers to a paraelectric-ferroelectric transition and not to ferromagnetism, but the syntactic structure is almost identical to what was found when describing the magnetic *T*_C_. Such ambiguity can also be found in constructions such as ‘The melting temperature of a compound marks the solid-liquid phase transition. This critical temperature for Fe is 1538 ^∘^C’. Since the classification is performed at the sentence level, the content of ‘This critical temperature for Fe is 1538 ^∘^C’ is evaluated independently from that of the preceding sentence. Then, it is expected to be erroneously classified. These limitations are inherent to working at the sentence level, and further effort is needed in order to resolve the issue effectively. Here, we mitigate the drawback by selectively analyzing scientific texts taken from the magnetism subject area.

**Table 1:** Performance of the three modules developed for the Curie temperature extraction: the sentence-level relevancy classifier, the NER, and the relation classifier.

Model	Entity	*P*	*R*	*F*_1_	Support	TrS	TeS
Classifier		0.83	0.80	0.81		3941	801
NER	Chem	0.92	0.86	0.89	754	1769	168
	*T*_C_	0.97	0.81	0.88	42		
Relation		0.72	0.64	0.68		200	50

Results are presented for the test sets. Here we report: precision, *P*, recall, *R*, and *F*_1_ score. The size of the test (TeS) and training (TrS) sets are also given (number of sentences used). For the case of NER, we report results for both chemical entities (Chem) and *T*_C_, as well as the support.

The second row of Table 1 presents the results for the named entity-recognition step of our automated extraction pipeline. The precision, recall, and *F*_1_ score of both the classified entities, compound and Curie temperature, are all consistently very high, indicating an excellent performance of the BERT model for token classification. This allows us to extract mentions of compounds and Curie temperatures from the sentences identified using the sentence-level relevancy classifier. Furthermore, given the context-aware nature of BERT-based language models, similar entities can be discriminated against, based on the syntactic and grammatical context in which they appear. In practice, our BERT model can, for the most part, differentiate between temperatures generally and those specifically mentioning critical temperatures. It should be noted, however, that this context-awareness suffers the same weakness as mentioned above in being limited in its ability to differentiate between different types of critical temperatures relating to phase transitions. Regardless, it performs excellently for the purpose of recognizing both compounds and Curie temperatures.

Relationship extraction has proved to be the most challenging task in our pipeline due to the sheer quantity of potential combinations of words in various syntactic structures. This also constitutes the major challenge when defining rule-based grammar constructions for methods such as ChemDataExtractor. The last row of Table 1 summarizes the key evaluation metrics for the BERT relationship-extraction model. Although this is the module presenting the lowest scores, the model still exhibits reasonably good performance and, therefore, it is useful to associate the correct compound-property pairs, thus improving the quality of the final database. In the remainder of this section, different alternative schemes for associating compounds and properties are compared with this relations classifier system. Furthermore, we will discuss the effect of considering Curie temperature values taken from phrases with multiple compound-property mentions on the integrity of the final extracted database.

Given the good performance of the three modules, a pipeline is constructed to automate the extraction of property values from unstructured literature. Approximately 180,000 full-text XML papers have been downloaded and split into lists of sentences. These sentences are subsequently run through the sentence-relevancy classifier, yielding a total of ~55,000 sentences considered relevant. Here, a sentence will be relevant, if it is likely to contain a Curie temperature mention. The resulting sentence list is passed through the named entity recognition system (NER-BERT). Sentences containing a single mention of a Curie temperature and a single compound are directly added to the database. When the sentences contain multiple references of compounds and/or Curie temperatures, then a list of sentences with all the possible pairs of entity mentions is built. These are then classified with the relationship classification BERT model. The compound/property pair predicted by the model to be correct is then added to the database.

The data extracted is subsequently post-processed by scaling all the temperatures to units of Kelvin and by filtering out compositions that could not be expressed as a combination of chemical elements (i.e., commercial or colloquial names for compounds). All chemical formulas are scaled to have normalized integer coefficients (e.g., Ga_0.5_Fe_2.5_O_4_ becomes GaFe_5_O_8_). The final extraction has given us a database containing 3518 distinct compound-property entries together with their digital object identifiers (DOI).

The same workflow is then executed for the case of the electronic band gap extraction task, and the performance metrics of each of the steps in our pipeline are summarized in Table 2. The performance of the sentence-level classifier, first row, is indeed excellent on the test set, with a perfect recall and a slightly lower precision at 0.95. These metrics are even higher than those found for the Curie temperature and indicate an almost perfect ability to differentiate between sentences that contain or do not contain information about the band gap of a compound. It is assumed that the superior performance of this classifier, even with fewer training examples, is due to the reduction in ambiguity in reporting band gaps, when compared to the likely similarities in syntactic structures that result from the reporting of critical temperatures. Similarly, the NER step consistently remains very performant, with a high precision, recall, and *F*_1_ score. Finally, we find that the relationship-extraction step for the case of the band gap outperforms significantly that of the Curie temperature. This is believed, once again, to be related to the more consistent way in which band gaps tend to be reported within the scientific literature, with the sentence structures generally more formulaic than in the case of Curie temperature mentions. This hypothesis is further highlighted by the improved performance of extraction methods relying on the rule associating compounds with band gaps based on the order in which they appear in the sentence. This enhancement in performance can be seen in Table 5 and is discussed further in Section II E.

**Table 2:** Performance of the three modules developed for the band gap extraction: the sentence-level relevancy classifier, the NER, and the relation classifier.

Model	Entity	*P*	*R*	*F*_1_	Support	TrS	TeS
Classifier		0.95	1.00	0.97		404	134
NER	Chem	0.80	0.96	0.87	1166	4000	1000
	Band Gap	0.78	0.97	0.87	119		
Relation		0.88	0.88	0.88		300	80

Results are presented for the test sets. Here we report: precision, *P*, recall, *R*, and *F*_1_ scores. The size of the test (TeS) and training (TrS) sets are also given (number of sentences used). For the case of NER, we report results for both chemical entities (Chem) and *T*_C_, as well as the support.

In this case, ~77,000 papers have been downloaded for the band gap extraction and split into lists of sentences. Running the sentence-level classifier yields a dataset of ~126,000 sentences deemed likely to contain a band gap. The post-processing steps are performed as previously described, scaling all units to eV in this case, yielding a final database of 2090 unique compound-property relationships.

### Comparison with rule-based methods

We now compare the performance of our BERT-PSIE workflow with the state of the art of rule-based methods. The test has been designed following the approach introduced in references^42,45^ and conduced over 200 manually annotated abstracts for both the compound-Curie temperature and compound-band gap extraction (200 each). The abstracts are taken from the arXiv dataset^46^ by running a keyword search on a sample of unused entries in the database. This means that the content of the test corpus has not been already included in any other sets used to construct BERT-PSIE. For the *T*_C_ the keyword used for the search is ‘Curie’, but we exclude abstracts containing the term ‘Weiss’. In contrast, the band-gap corpus is constructed using a keyword search for abstracts containing any of the terms ‘band gap’, ‘band-gap’, or ‘bandgap’, alongside the term ‘eV’. Random abstracts are sampled from the resulting corpus to yield a dataset of 200 for both cases. This selection has the aim to increase the number of positive extraction targets (support).

Both BERT-PSIE and ChemDataExtractor are run on this test set. A record in the extracted database will be deemed true-positive only if all entities in the target compound-property pair are present and matched to the manual annotation. The number of true positives, false positives, and false negatives for the extraction tasks are manually counted for each property extraction, allowing for the calculation of the precision, recall, and *F*_1_ score for each model. The ChemDataExtractor model for the extraction of Curie temperature came from the same rule-based pipeline reported in ref. ^42^. It has not been possible to include the snowball model of this extraction pipeline to make it fully hybrid, as the model used in the work is not readily available. In the case of the band-gap extraction with ChemDataExtractor, however, the full hybrid extraction is performed using the model from ref. ^45^. The results of this comparison are presented in Table 3.

**Table 3:** Performance of the extraction carried by BERT-PSIE and the rule-based ChemDataExtractor performed on the same corpus.

	*T*_C_			Band gap		
Model	*P*	*R*	*F*_1_	*P*	*R*	*F*_1_
ChemDataExtractor	0.67	0.49	0.56	0.68	0.55	0.61
Single Mentions	0.82	0.20	0.32	0.78	0.23	0.35
BERT-PSIE	0.67	0.31	0.42	0.70	0.40	0.51
BERT-PSIE + ChemDataExtractor	0.64	0.64	0.64	0.63	0.72	0.67

The comparison is executed on 200 annotated abstracts for each one of the tasks, namely the *T*_C_ and band-gap extraction. The precision, recall, and *F*_1_ score are presented for BERT-PSIE (single mentions only and the full pipeline), ChemDataExtractor, and the combination of the two methods. The manually annotated datasets have a support of 45 entries for *T*_C_ and 109 entries for the band gap.

From the table, it is clear that the precision of both models is very consistent across both quantities, with the full BERT-PSIE pipeline slightly outperforming the hybrid ChemDataExtractor model in the case of the band-gap extraction. In order to isolate the impact of the relationship-extraction module and to estimate the amount of noise that this introduces, we also consider the case where we only extract data from sentences containing a single compound-property relationship. This means excluding sentences where multiple *T*_C_’s are associated with a list of compounds, sentences that are prone to intrinsic ambiguity, since, in natural language there are many different semantic ways to relate two lists of quantities. We denominate this case as ‘single mentions’, for which we observe a significant increase in the extraction precision. However, this gain in precision is compensated for by a severe reduction in the recall, thereby reducing the overall *F*_1_ score. The BERT-PSIE pipeline results are more selective in the data extracted, as any potential contextual ambiguity in reporting a given value is far more likely to deter a context-based system than a rules-based one. This fact results in a general decrease in the BERT-PSIE recall, when compared to the rules-based and hybrid pipeline. One can then argue that recall is generally a less important metric for the fidelity or usefulness of the resulting database. This point is further investigated by introducing additional metrics measuring the real-use case of the extracted data. Their goal is to evaluate the usefulness of the resulting automatically generated database. This discussion is reported in sections II E and II F.

The final evaluation for both targets, is performed over the combination of the values extracted with BERT-PSIE and ChemDataExtractor (last row of Table 3). This returns a sharp increase in the recall when compared with either of the methods. The implication of this result is that the rules-based pipeline and the BERT-based pipeline have different strengths in extracting quantities. In fact, the distinct increase in recall implies that there is quite a little overlap in the quantities extracted by the two methods. Then, the reduction in precision relative to the individual BERT-PSIE and ChemDataExtractor is a consequence of both the true-positive entries in common to the two methods are not doubly counted, but any incorrectly extracted values are added to the total database. This leads to an increase in the false positives relative to the true ones, and hence a reduction of the total precision.

### Extraction performance and database structure

For this section, our discussion begins with the case of the Curie temperature extraction. Evaluating the metrics (precision, recall, and *F*_1_ score) of a data-extraction method provides only limited information on its performance in that it gives only general indications of the quality. Clearly, the ultimate test is set by its success in the extraction task it has been designed for, namely by the quality of the data extracted and by their potential use in downstream tasks (e.g., the construction of ML models). Performing such an assessment is generally challenging since one lacks manually curated data that are difficult to assemble because of the elevated time investment involved. In our case, the situation is much more favorable, since we can compare our automatically extracted data with manually curated databases from various sources. In the case of the Curie temperatures, we avail of the database of Nelson et al.^18^. This dataset has been created by aggregating the *AtomWork* database^47^, *Springer Materials*^48^, the *Handbook of Magnetic Materials*^49^ and the book *Magnetism and Magnetic Materials*.^50^. Nelson’s database is then combined with *T*_C_ values from a dataset manually aggregated by Byland et al.^41^, which is mainly focused on, although not limited to, Co-containing compounds. Thus, this combined database is considered to be our ground truth, which amounts to 3,638 unique ferromagnetic compounds and their associated Curie temperatures.

Our results are also compared with a second database, namely the one obtained by combining the rules-based ChemDataExtractor scheme with a semi-supervised snowball algorithm^42^. At this point, it is important to remark that the two automatic-extracted databases are based on a rather similar corpus of papers, namely those obtained with a keyword search from Crossref. This search includes relatively recent articles and information is extracted only from the text. In contrast, the database of Nelson et al. is largely based on data reported in tables and includes much historical information (results published as early as the fifties). Despite the similarity in their respective corpora, however, the BERT-PSIE and ChemDataExtractor databases, of several thousand data points each, present a remarkably small overlap of only 694 compounds. Of similar size is the overlap between the automated and manual datasets of 687 compounds for BERT-PSIE vs manually curated and 595 for ChemDataExtractor vs manually curated. Overall the three datasets (BERT-PSIE, ChemDataExtractor, and the manually curated one) share only 262 compounds. All comparisons performed in this section are made by taking the median Curie temperature value for compounds that contain multiple entries for each dataset.

**Fig. 2:**
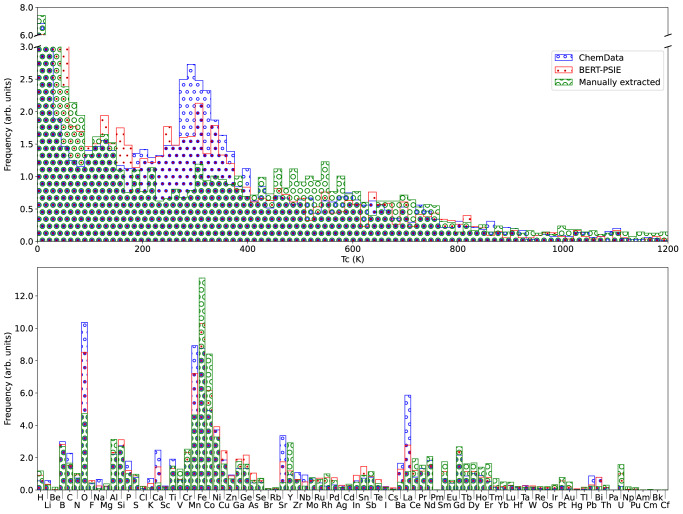
Comparison between the content of the different databases: (red box) BERT-PSIE, (blue box) ChemDataExtractor, and (green box) the manually extracted database of ref. ^18^. **a** Normalized distribution of the Curie temperatures extracted. A peak is visible in the distribution of ~300 K in both the autonomously extracted databases, which is not seen in the manually extracted one. **b** Relative elemental abundance across the compounds present in a database. Although there is general agreement among the three databases, additional peaks are observed for various elements in the case of automatically extracted data, which are not present in the manually curated dataset. The most severe of these discrepancies is in the relative abundance of Mn- and O-containing compounds. Note that the automatically extracted datasets and the manually curated ones are based on different literature libraries.

Before going into the details of the comparison, we observe that a significant source of error is associated with elemental compounds (e.g., Fe, Co, Ni, Gd, etc.), where the error is the variance of the extracted *T*_C_ compared with our ground truth. This is due to the general difficulty of the NER in differentiating between an elemental compound and an element used as a dopant in an otherwise non-magnetic material (e.g., bulk Mn vs Mn-doped GaAs). As dopants can appear in a multitude of concentrations and in a large variety of hosts, erroneous assignments may result in a large spread in the distribution of the temperatures collated. With this exception, the distributions of Curie temperatures across the different databases are in very good agreement with each other, as one can see in the top panel of Fig. 2. The agreement is particularly close between our automatically extracted dataset and the one constructed with ChemDataExtractor, but both present a peak in the distribution at around room temperature, which is absent in the manually curated one. There are two possible reasons behind this feature: either there is a bias in the most recent literature towards critical temperatures close to 300 K, or errors in the model aggregate *T*_C_ values close to ambient temperature. In support of the second hypothesis, it is worth recalling that mentions of room temperature feature heavily in sentences containing the target information, even if the room temperature is not the target temperature. An example of this situation is the sentence: ‘The magnetization curve at 300 K was obtained and the Curie temperature was determined by TGA under a magnetic field, yielding a Curie temperature of 1043 K for Fe.’ Despite these differences, the still good similarity between the Curie temperature distributions indicates that the relative abundance of high- and low-temperature ferromagnetic materials has been adequately captured by our automated extraction technique without the need for complex grammar-rule definitions.

Further understanding can be achieved by looking at the relative elemental abundance across the unique compounds present in a given database (the frequency at which a particular element appears in the database). This is shown in the lower panel of Fig. 2, again for all three datasets. As expected, the largest abundances are found for the magnetic transition metals, some of the rare earths, and oxygen, a feature shared by all databases and corresponding to the actual elemental distribution among magnets. More interestingly, it appears that the automatically compiled databases overestimate the presence of Mn and O and that of di- and tri-valent alkali metals (Ca, Ba, Sr, and La). Such an overestimate with respect to the manually extracted dataset is significantly more pronounced for the ChemDataExtractor data than for the ones obtained with our workflow. It is likely that such a difference in distributions is mainly attributable to the data primary sources, which are different in the case of manually and automatically curated datasets. In particular, the most recent literature used in our extraction and in that performed by ChemDataExtractor, contains many entries related to Ca-, Ba-, Sr- and La-containing perovskites (e.g., manganites).

**Fig. 3:**
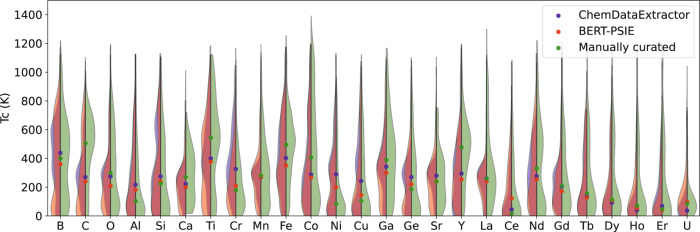
Comparison between the *T*_C_ distributions of compounds containing the most common elements found in ferromagnets. The violin plots display the *T*_C_ distribution of the compounds containing specific elements in the dataset automatically generated with BERT-PSIE (red) and ChemDataExtractor (blue), and in the manually curated ground truth (green). Only the most common elements appearing in the datasets are displayed here. The dots show the median of each distribution.

The influence of the primary data source on the final dataset is further confirmed by comparing the *T*_C_ distributions of compounds containing the 25 most common elements, which is presented in Fig. 3. Generally, there is excellent agreement between the distributions of the two automatically extracted datasets, which contain entries extracted from similar sources. Then, BERT-PSIE generally captures a similar distribution to the manually extracted values, although there are evident discrepancies for certain elements. This may be an indication of the historical change in research focus between the sources used for the ground truth compared with the sources for the automatically extracted cases.

**Fig. 4:**
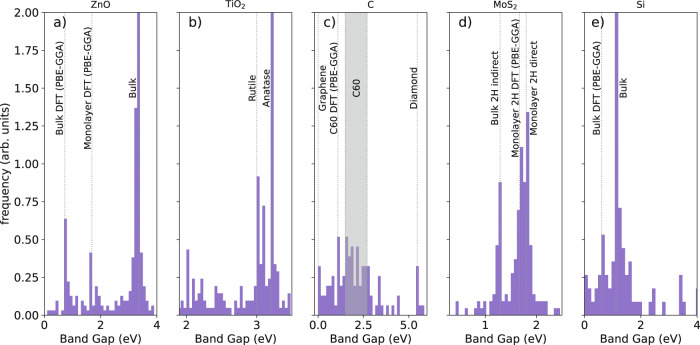
The distribution of band-gap values for the five most common chemical formulas found in the database of band-gaps generated by BERT-PSIE. The histograms report the relative abundance, while dashed lines indicate gap energies corresponding to specific experimental measurements or theoretical calculations.

A similar study, with similar results, is performed on the distribution of the extracted band gaps, which can be found in the Supplementary Information (SI) (see Supplementary Fig. 3). As for the case of the Curie temperatures, we find strong similarities between our database and the one obtained from ChemDataExtractor, but both present some level of disagreement with the manually curated one. We now discuss the origin of such disagreement by looking at the extracted band-gap distributions, see Fig. 4, of the five most common chemical formulas in the database, namely ZnO, TiO_2_, C, MoS_2_, and Si. The most interesting feature is that while there is a spread of band-gap values for all five compounds, these are not uniformly distributed. In contrast, the band gap densities seem to have a clear peak structure, with multiple high-frequency values appearing. This can be attributed to different means of obtaining the band gap of a material (experimental optical, experimental transport, theory, etc.), and to different polytypes, structures, or dopant-varied compounds.

Going into more detail, consider first the case of ZnO in Fig. 4a. In the distribution, we find three clearly visible peaks, which are easily associated with the experimental bulk band gap (3.37 eV^51^), the density-functional-theory (DFT) calculated one for bulk ZnO (0.73 eV^51^ for PBE-GGA) and the DFT-calculated one for monolayer ZnO (1.69 eV^52^ again PBE-GGA). A similar situation is encountered for Si in Fig. 4e, where the two main peaks are attributed to the experimental bulk indirect gap (1.1 eV^53^) and the one returned by DFT simulations (0.61 eV^2^, PBE-GGA). A different situation is then encountered for TiO_2_ in Fig. 4b, where the two main dominant peaks both correspond to experimental gaps, but they are for two different polymorphs, namely anatase (3.2 eV) and rutile (3.0 eV)^54^. Finally, MoS_2_ and C in Fig. 4d, c display more complexity. In the first case, we find three dominant peaks. In fact, together with the experimental bulk indirect band gap of 1.29 eV^55^, many mentions in the literature concern the experimental band gap of the monolayer form of MoS_2_ (1.8 eV^56^) and the DFT estimate of the same (1.67 eV^57^, PBE-GGA). Carbon, in contrast, deserves attention on its own since a large variety of polymorphs is possible. In fact, the distribution shows a clear peak for semimetal graphene^58^ and one for the bulk diamond structure (5.47 eV^59^). Then, there is a uniformly distributed region, which is characterized by band gap values associated with carbon buckminsterfullerenes, C60. This extends over the 1.5–2.7 eV range, and has a clear peak at the DFT value of 1.09 eV (PBE-GGA)^60^.

### Database quality for downstream tasks: Curie temperature

**Fig. 5:**
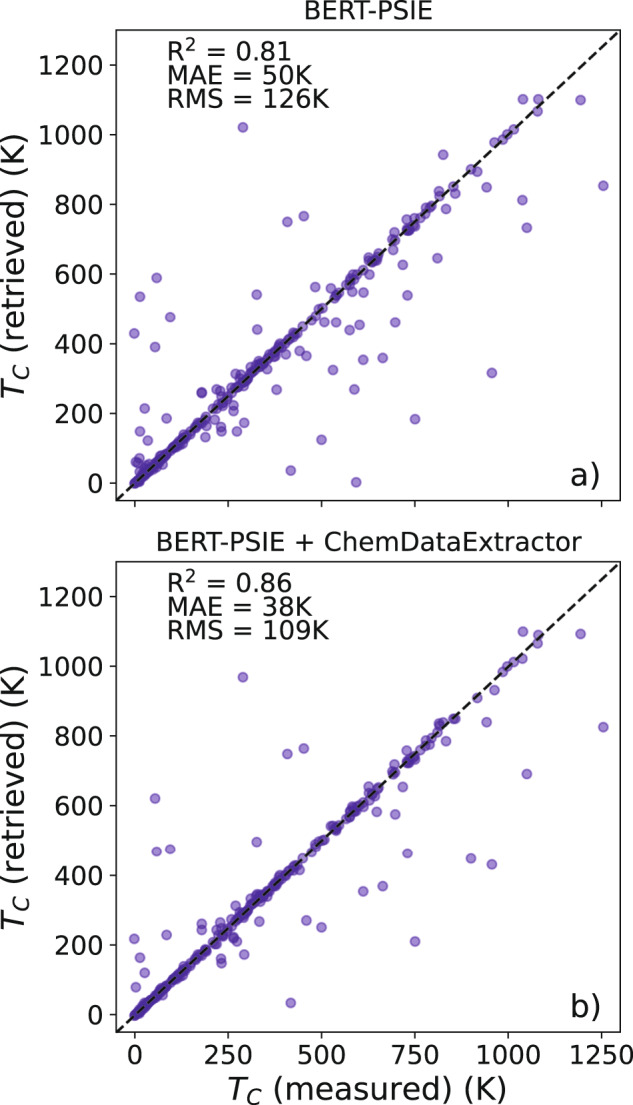
Query-test for the BERT-PSIE-generated *T*_C_ dataset. **a** Comparison between the *T*_C_ queried in the dataset automatically generated by BERT-PSIE and the values contained in the manually curated dataset. The comparison is performed over the 262 compounds that are shared by all datasets examined in this work. The median value is returned whenever multiple *T*_C_ values are collected for a given compound. **b** The same comparison is performed on the dataset resulting by combining the one generated by BERT-PSIE and the one generated by ChemDataExtractor.

The quality of a material-property database can be quantified in terms of its usefulness for material design. Once again, we start the discussion from the Curie temperatures, by first assessing that the returned value to a query related to a compound present in the database is reliable. To achieve this goal, we have designed a ‘query test’ comparing the Curie temperatures automatically extracted with the one present in our reference manually extracted dataset. In order to make the comparison between our database and the ChemDataExtractor-generated one not dependent on the particular class of compounds extracted, we only compare entries that are shared by all the datasets (262 compounds). The query test results for BERT-PSIE are reported in Fig. 5, while the performance metrics for the different datasets are summarized on the left-hand side of Table 4.

**Table 4:** Performance comparison between the different automatically generated *T*_C_ datasets against the manually curated one from refs. ^18,41^.

	# Entries	Query			RF predictions		
		*R*^2^	MAE (K)	RMSE (K)	*R*^2^	MAE (K)	RMSE (K)
ChemDataExtractor	4289	0.78	**48**	137	0.65	**123**	176
This work							
Single mentions	1858	0.77	51	139	**0.66**	128	**174**
Order of appearance	2682	0.77	51	141	0.65	126	176
All combinations	4308	**0.81**	52	127	0.61	134	184
BERT-PSIE	3518	**0.81**	50	**126**	0.65	126	**174**
BERT-PSIE + ChemDataExtractor	7052	0.86	38	109	0.69	118	165

The left-hand side of the table refers to the query test, while the right-hand side refers to the RF *T*_C_ predictor. Together with the BERT-PSIE and ChemDataExtractor databases, we also consider different BERT-assembled datasets obtained by using different relation-classification strategies (see details in the text). The query benchmark is done over the 262 compounds that are shared by all the datasets, while the RF predictions are done over 2623 compounds that are not present in any of the automatically collated datasets. Values for the best-performing datasets are in bold. Additionally, the last line of this table shows the performance on these tests for the combined databases generated by BERT-PSIE and ChemDataExtractor, leading to the best results.

As mentioned before, the most challenging step in our extraction workflow is the relation-classification step. In order to evaluate the performance of our model for relation classification, a variety of additional extraction strategies have been attempted and compared against the ground truth dataset. The first of these strategies involves, as before, taking only the ‘single-mention’ results extracted from sentences containing only a single mention of a compound and a single mention of a Curie temperature value. In this case, we assume that the two entity mentions are related to each other, thus removing the need for any relation-assignment step (‘Single Mention’ in Table 4). The second strategy imposes a rule that associates compound/value pairs based on the order in which they appeared in the text (‘Order of Appearance’ in Table 4). Finally, we have taken every possible combination of compound/value pairs in order to compare our results with random associations (‘All Combinations’ in Table 4). This choice corresponds to a relation-classifier model that always outputs a positive classification. Table 4 is complemented by results obtained with our constructed BERT classifier (‘BERT-PSIE’), with the data extracted by ChemDataExtractor and by aggregating these last two datasets (‘ChemDataExtractor + BERT-PSIE’).

As can be seen from Table 4, all of the datasets compiled with our rule-free pipeline have metrics comparable to those of ChemDataExtractor, and the one constructed with BERT-PSIE appears to be the best-performing on almost all the query-test metrics. In particular, BERT-PSIE returns the best *R*^2^ coefficient of 0.81 and root mean squared error (RMSE) of 126 K. Interestingly, BERT-PSIE gives us a mean absolute error (MAE) slightly larger than that obtained with ChemDataExtractor. This suggests that BERT-PSIE achieves an accuracy on-par with ChemDataExtractor (the two produce datasets equally similar to the manually curated one), but it is slightly less prone to display large outliers. Note, however, that the notion of an outlier and its relevance to the overall performance of a method need to be taken with some caution in this query test. In fact, if the *T*_C_ of a compound is erroneously extracted (the ML model extracts the wrong temperature), there is no particular advantage of having the wrong *T*_C_ close to the real one. This point can be better understood by looking at the parity plot in Fig. 5, where entries are either on the parity line (exact extraction) or away from it without any particular correlation with the actual *T*_C_ value (erroneous extraction).

In any case, the fact that BERT-PSIE performs better than any other BERT-based models using different relation-classifier strategies demonstrates that the inclusion of a context-aware mean of extracting compound-value pairs from literature is advantageous. Unfortunately, this is not a massive improvement, since the metrics are rather close to those obtained by considering randomized combinations of all possible compound-value pairs (‘All Combinations’). Indeed, more sophisticated methods to establish the correct compound-property associations will help in producing a better-automated dataset.

Our second test probes the ability of a given data-extraction strategy to create datasets of sufficient quality to enable the construction of predictive ML models. In practice, we want to establish whether the data extracted can be the platform for models that predict the *T*_C_ of unseen compounds. In particular, we target compositional models, namely ML algorithms using information directly accessible from the chemical composition of a given compound as features. For the case of the Curie temperature associated with the ferromagnetic transition, in fact, it has been shown that compositional models can achieve good performance if trained on manually curated data^18^ (note that the model mentioned does not describe other magnetic phases, e.g., antiferromagnetic structures). In order to test the ability of a dataset to function as a reliable data platform for ML models, we train on each automatically generated dataset a random forest (RF) model that takes as input compositional features, as done in refs. ^18,61^. We have chosen the same input features for all the RF models trained, since, in all the cases considered, we have not observed any improvement when adding more features. We then compare the predictions on a set of compounds that are not present in the training set with the values extracted manually from the literature. For this test, we consider predictions over 2623 compounds for which we have a manually extracted *T*_C_ that does not appear in any of the datasets automatically extracted. With this choice, both our tests are performed on the same compounds for all the datasets considered. For compounds with multiple values of extracted *T*_C_, the median value of the collated results is taken as the associated Curie temperature, according to the procedure introduced in ref. ^18^. We have also tested other summary statistics, such as the mean and the mode, without finding any significant difference in the results.

Again the results of our test are reported in Table 4 (right-hand side), where one can clearly see that BERT-based extraction workflows perform rather similarly to the established rule-based method. In particular, the full workflow, BERT-PSIE, has an *R*^2^ identical to that obtained by ChemDataExtract, with a better RMSE but worse MAE, we observe for this second test a result similar to that found for the query test. Most interestingly, we find that the inclusion of entries extracted in conjunction with the relations-classification step in the database does not improve the performance of the predictor. In fact, using single mentions only returns us the better *R*^2^ value of 0.66 and an RMSE of 174 K, while BERT-PSIE gives us a slightly degraded *R*^2^ at 0.65 and an identical RMSE, although it slightly improves the MAE (by ~2 K). This is possibly due to the fact that the inclusion of the entries from multiple mentions inherently adds noise to the database. Thus, despite the fact that the model can be trained over a much larger dataset, no significant improvement is detected.

**Fig. 6:**
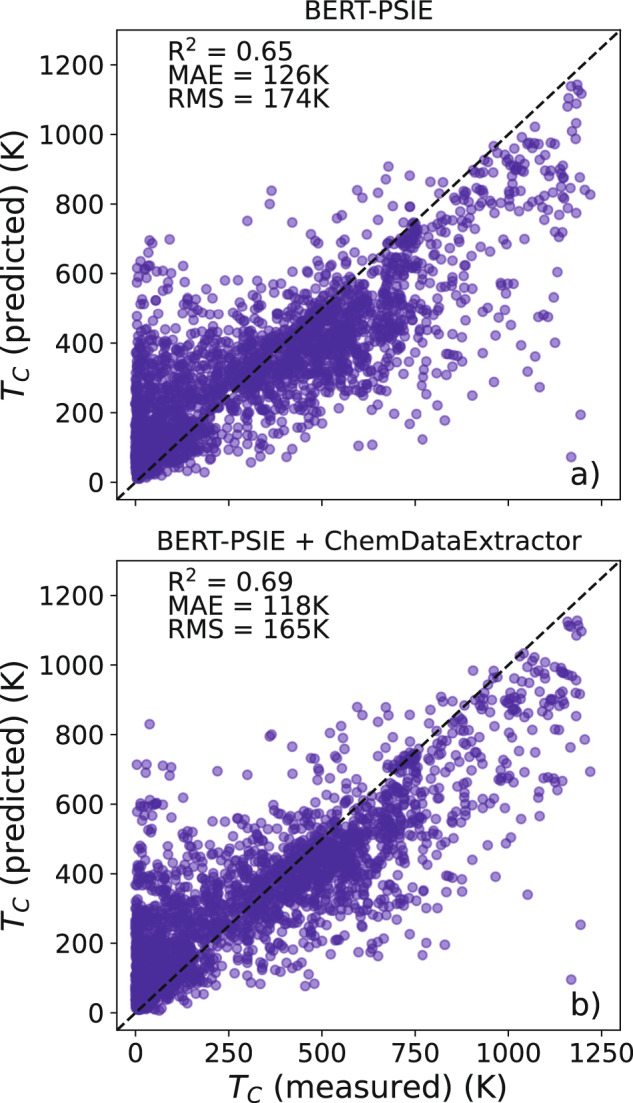
Performance of a random-forest model for *T*_C_ trained over automatically extracted databases. Parity plot (predicted *T*_C_ vs manually extracted *T*_C_) for the best RF compositional model constructed **a** on the BERT-PSIE dataset and **b** on the combined BERT-PSIE and ChemDataExtractor dataset. The test set consists of the 2623 compounds that are not present in any of the automatically generated datasets considered in this work but for which we have a *T*_C_ manually extracted from the scientific literature.

The parity plot of our best RF model trained on the full BERT-PSIE dataset is presented in Fig. 6. In general, the *T*_C_ trends are captured, but it is also clear that the model is significantly inferior to that presented in ref. ^18^, which reports a MAE of 57 K. This is roughly a factor of two smaller than the 126 K obtained for a random forest model trained on the data extracted with BERT-PSIE. Although this can be partially attributed to noise in the data, for instance, to the likely presence in the BERT-PSIE dataset of critical temperature associated with antiferromagnets, one also has to consider that the data used in ref. ^18^ were highly curated even after the collection. For example, additional data on paramagnets was included to improve the low-temperature part of the distribution, while data corresponding to different concentrations of metallic alloys was selectively excluded to better balance the chemical distribution. All these post-processing steps were not performed here, since our task is simply that of assessing the quality of the automatically compiled dataset. In fact, one expects that automatically constructed datasets can reach sizes large enough for such post-processing steps to not be necessary.

Given the fact that the overlap between our BERT-PSIE dataset and the one generated by ChemDataExtractor consists of only 694 compounds, we have constructed an additional dataset resulting from the combination of the two. This combined database contains 7052 distinct entries and performs best on all the metrics evaluated in each test (see the last line of Table 4 and the bottom plots in Figs. 5 and 6). The improvement in performance is likely due to the much larger size of the dataset (approximately double the original two) and the corresponding reduction of the noise present in the median values. When tested against RF models, the much larger number of compounds allows for a better sampling of the chemical space, resulting in more accurate predictions. As it stands, this combined dataset represents the best database available for ferromagnetic *T*_C_, automatically extracted from scientific literature according to the test designed here.

The implication of this is that the quality of automatically extracted databases improves markedly with an increase in the number of disparate sources used in the extraction. There is also an argument to be made that a combination of rules-based and rule-free methods may be the best-performing strategy for automated extraction, as seen in Table 3.

A similar study was conducted with respect to the extractions performed by ChemDataExtractor over the same sentences deemed relevant by the BERT-PSIE sentence classifier module. The results can be found in the Supplementary Discussion and reinforce the similarity in performance of the two approaches.

**Fig. 7:**
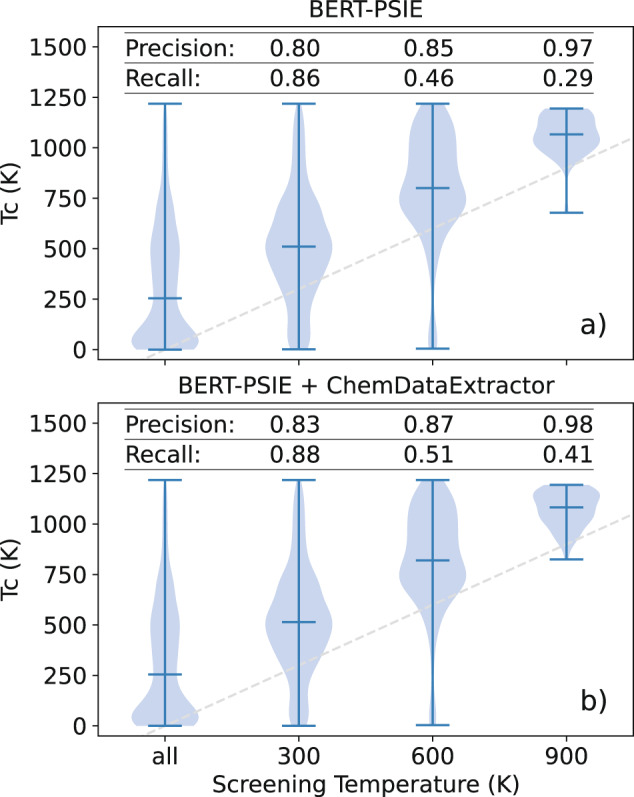
Evaluation of the RF models constructed on automatically extracted datasets as classifiers. **a** Violin plots showing the *T*_C_ distributions of the compounds screened using an RF model trained on the BERT-PSIE data and compared with the manually extracted values. The dashed line is the parity line highlighting how the median of the screened distribution increases as the screening threshold increases. Despite a low recall, the precision is high enough to select compounds likely to have a *T*_C_ higher than a given threshold. The screening is done on compounds not present in the training set of the RF. **b** The same test is performed by training an RF model on the combination of the BERT-PSIE and ChemDataExtractor datasets.

To conclude, as a final evaluation of the usefulness of the extracted database, we test the ability of the RF model trained on the BERT-PSIE dataset to screen unseen compounds with respect to a certain *T*_C_ threshold. This test attempts to simulate a common use case for such ML models. Note that typical magnets employed as part of some room-temperature technology (e.g., data storage, electrical motors) need to have a *T*_C_ above 600 K, so that classifying magnets according to such a threshold is of significant technological relevance. We used the RF model trained on the automatically generated dataset to predict if magnets have a critical temperature exceeding 300 K, 600 K, and 900 K, respectively. The test set for this predictor is constructed from compounds that are present in our manually curated dataset, but not in the one generated by NLP. The results of this screening, compared with the distribution of the true *T*_C_ of these compounds can be seen in Fig. 7. The shaded blue area of Fig. 7 represents the distribution of values predicted to have a *T*_C_ greater than the dashed line, representing the screening temperatures (300 K, 600 K, and 900 K, respectively). While the recall of this screening is quite low, the high precision biases the initial distribution into sets with higher and higher *T*_C_, thus demonstrating the usefulness of the extracted database in screening for compounds with *T*_C_ above a desired threshold. The low recall means that certain compounds with *T*_C_ above the desired temperature will not be predicted to be in the set of compounds above the temperature, however, the compounds predicted to be in this set can be trusted with high accuracy to have a *T*_C_ above the desired threshold. Training a model using the combined datasets from BERT-PSIE and ChemDataExtractor results in a higher screening recall (see Fig. 7). This provides an example of how the utility of these automated databases can be improved both by expanding the corpus size used for extraction and by introducing new extraction techniques.

### Database quality for downstream tasks: band gap

To further validate the performance of BERT-PSIE, a similar study is performed, now with the target being an aggregated dataset of compounds and their associated band gap. For the manually curated test set in this instance, a database of band gaps from reference^20^ is utilized and compared with our results and with the results of the hybrid ChemDataExtractor model from Dong et al.^45^, when run on the same literary corpus. The original dataset from this paper is not used as a direct comparison between the models, since the workflow implemented in ref. ^45^ also separately processes tables, which are not considered by BERT-PSIE. The results of the comparison between the two methods on the same corpus can be seen in Table 5. In this case, the BERT-PSIE pipeline outperforms or equals the hybrid ChemDataExtractor method by every metric while extracting a very similar number of unique compound-band gap relationships. Interestingly, for sentences containing multiple mentions, the best strategy to sort out relations seems to be the order of appearance, which outperforms all other methods. This is in contrast with the degradation in performance reported for the case of Curie temperature, Table 4, pointing to an intrinsic difference in the way these two quantities are reported in natural language. It then appears that reporting the band gap is far more procedural than reporting the Curie temperature, thus the use of a more sophisticated method of establishing the correct associations between compounds and properties introduces a source of noise. This result is clearly property-dependent, but it is also fair to note that the difference in performance is only marginal.

**Table 5:** Performance comparison between the different automatically generated band gaps datasets against the manually curated one from ref. ^20^.

	# Entries	Query			RF predictions		
		*R*^2^	MAE (eV)	RMSE (eV)	*R*^2^	MAE (eV)	RMSE (eV)
ChemDataExtractor	2185	0.54	0.78	1.34	0.59	**0.62**	0.87
This work							
Single mentions	1,246	0.65	0.67	1.17	0.61	**0.62**	0.85
Order of appearance	1819	**0.67**	**0.64**	**1.13**	**0.62**	0.63	**0.84**
All combinations	2581	0.63	0.71	1.21	0.60	0.63	0.86
BERT-PSIE	2021	0.64	0.67	1.19	0.61	**0.62**	0.85

The left-hand side of the table refers to the query test, while the right-hand side refers to the RF band gap predictor. Together with the databases constructed using BERT-PSIE and ChemDataExtractor, we also consider different BERT-assembled datasets obtained by using different relation-classification strategies (see details in the text). The query benchmark is done over the 231 compounds that are shared by all the datasets, while the RF predictions are done over 2046 compounds that are not present in any of the automatically collated datasets. Values for the best-performing datasets are in bold.

Finally, the parity plot for the query test and that for an RF model for band-gap predictions are shown in the Supplementary Figure. The results of the query test are similar to those found for the Curie temperature, although it seems that a more diffuse distribution of band gaps is now observed. This is associated with the ambiguity in the various band-gap definitions noted before (e.g., the case of C), the ambiguity that is less relevant for the *T*_C_. The RF model, instead, appears to have a slightly inferior *R*^2^ than that constructed for the *T*_C_, but benchmarks similarly with models that can be constructed on manually curated data. In fact, we obtain an MAE of 0.62 eV, against the value reported on MatBench^62^ of 0.33 eV, for the best-performing model trained on the same dataset.

## Discussion

We have proposed a workflow to automatically extract structured data from unstructured scientific literature. This has minimal need for an extensive implementation effort and little or no requirement for familiarity with complex grammar-rule definitions and natural language processing. We have then shown some possible use cases, demonstrating the ability to generate a database of ferromagnetic Curie temperatures and electronic band gaps comparable to the one generated using ChemDataExtractor, the state-of-the-art rule-based method for data mining from the scientific literature. This work opens the door for rapid and easy access to experimental-property databases for materials informatics applications.

Crucially, we have carefully benchmarked the constructed databases against manually curated reference ones through extensive query tests, which is a step particular to this work. These have allowed us to critically assess the benefit of certain design choices in our workflow, such as the relation-extraction step. Most importantly, we have been able to understand where improvements can be made and whether these are general or specific to the physical property extracted.

Finally, we have tested whether our automatically extracted databases of compounds and their properties can be used as a platform for constructing machine-learning models, namely whether the database quality is sufficiently high for integration in a material-discovery workflow. We have found that the mean absolute error of chemical-informed random-forest models, constructed over the automatically extracted database, is always larger than that achieved with manually curated ones, roughly by a factor two. Our best results have been obtained for a Curie temperature database combining data from rule-free (BERT-PSIE) and rule-based (ChemDataExtractor) methods, owing to the larger data volume and enhanced diversity. Notably, no manual curation was performed on the automatically extracted datasets, a fact that may be responsible for the differences in performance. In contrast, we have shown that our BERT-PSIE dataset is sufficient to construct machine-learning classifiers able to identify high-*T*_C_ magnets with high accuracy. This fact, together with the possibility to expand the dataset with the minimum effort offered by a rule-free strategy, suggests that natural-language-processing information retrieval can become an important asset in any material-discovery pipeline.

## Methods

### Human and numerical effort

All the BERT models used in this work have been fine-tuned on a single Nvidia A100 GPU accessed with Google Colab, with each model requiring <30 minutes for fine-tuning. In particular, we use an early stopping strategy based on the validation-set loss (more details are provided in Section I of the SI. As it stands, the time bottleneck in adapting the workflow to a new task remains related to the manual labeling of the text necessary for fine-tuning. All in all, this takes ~1 week. We now describe in more detail the algorithms and the training strategy used for the various models.

### Literature extraction

The Crossref REST API is used to execute a keyword search over all literature published by Elsevier. This yields metadata filtered to ensure that the full-text version of the paper is available for the purpose of text data mining. The metadata includes both abstracts and download links to the full-text papers. In the case of the Curie temperature extraction, the strategy used to build the training set required for the fine-tuning of the different BERT models starts from the collection and the manual labeling of 800 abstracts containing the term ‘Curie temperature’ from this initial search. We run the Natural Language Toolkit^63^ (NLTK) sentence tokenizer on these abstracts and label the tokenized sentences, which reference a Curie temperature, as relevant and the ones that do not as irrelevant (step 1 in Fig. 1). This step yielded a database of ~5000 sentences of which 189 are labeled relevant. The labeled dataset is used to fine-tune a BERT classifier model to find relevant sentences (i.e., sentences likely to contain a mention of the Curie temperature). The classifier extracts relevant sentences from the corpus of the papers. We then manually labeled 200 relevant sentences extracted from the corpus of the papers whose abstract was used in the previous step. These extracted sentences are manually labeled as described in step 3 of Fig. 1 and are then combined with the labeled abstracts. This combined corpus is utilized to fine-tune a BERT model for Named Entity Recognition (NER-BERT).

In the case of the electronic band gap, a slightly different strategy is developed for aggregating the necessary training data. The arXiv metadata is downloaded from the Kaggle dataset^46^ and an initial corpus of 1000 abstracts for annotation is constructed by searching the text of the corpus of abstracts for the terms ‘band gap’, ‘bandgap’, or ‘band-gap’. Contained in these 1000 abstracts were 171 sentences that contained band-gap values and thus were considered relevant. A sample of 501 sentences that contain no mention of band gap, and thus are considered irrelevant, was added to the relevant ones. This enables the creation of a dataset for the classifier of 672 sentences, which was split into a training, validation, and test set of 404, 134, and 134 entries, respectively.

The corpus used for the automatic extraction procedure of the Curie temperature (band gap) is obtained by executing a keyword search using the Crossref API for instances of the term ‘magnetic’ (‘electronic’). This yields a database of ~180,000 (77,000) full-text URLs of papers likely to contain a mention of a Curie temperature (band gap) value. The papers are then automatically downloaded and parsed into a list of sentences. A corpus of relevant PDF documents is also converted into plain text using PDFminer^64^ and is similarly parsed into sentences, which are concatenated to the same list. A list of candidate sentences likely to contain the desired material property information is then extracted using the BERT classifier from these corpora, yielding a total of around 55,000 sentences that are deemed relevant for the Curie temperature and around 126,000 for the electronic band gap.

### Relation extraction

The final step of our workflow for the automatic extraction of data consists of the identification of mentions of chemical compounds and the associated property (Curie temperature or band gap) in all the sentences classified to be likely to contain such information. This task is performed by the NER-BERT model (step 3 of Fig. 1), which is described here for the Curie temperature (the same applies to the band gap). For the sentences predicted by the NER-BERT model to have a single mention of chemical compound and a single mention of Curie temperature, we assume that the two quantities are related, and we add them to the database (step 5 in Fig. 1). If a sentence contains multiple mentions of chemical compounds and/or several Curie temperatures, the compound-temperature association will become ambiguous. This ambiguity is not uncommon in scientific literature, where one can find sentences like ‘the Curie temperature of Fe and Co are 1043 K and 1394 K, respectively’. Although easy to resolve for a human reader, semantic ambiguity becomes a problem for NLP. Here, we treat the problem as a relation classification task. In practice, following the approach of Soares et al.^65^, we fine-tune a BERT architecture to classify whether a pair of entities in a sentence is related by the “has a *T*_C_ of” relation.

The dataset needed for the fine-tuning is generated by sampling 100 sentences from among those predicted to contain multiple mentions by the NER-BERT model. For each sentence, all the possible pairs of compound-*T*_C_ mentions are considered one by one, and entity markers are added at the beginning and at the end of each entity mention. For example, from a sentence containing two chemical compounds and two Curie temperatures, we generate four sentences in which a different pair of entities is surrounded by entity markers. We use the markers [*E*1_start_], [*E*1_end_] to identify the compound mentions considered and [*E*2_start_] and [*E*2_end_] to identify the Curie temperature mention. Thus, by taking as an example the sentence, ‘The Curie temperature of Ga_0.5_Fe_2.5_O_4_ and Ga_0.7_Fe_2.3_O_4_ have been found to be equal to 413 ^∘^C and 347 ^∘^C, respectively’ (see Fig. 1), we construct the following four associations: (1) Ga_0.5_Fe_2.5_O_4_ and 413 ^∘^C, (2) Ga_0.5_Fe_2.5_O_4_ and 347 ^∘^C, (3) Ga_0.7_Fe_2.3_O_4_ and 413 ^∘^C, (4) Ga_0.7_Fe_2.3_O_4_, and 347 ^∘^C. Then, the sentences with the marked pairs generated are manually labeled for a binary classification task, where we will deem a sentence positive, if the relation “has a *T*_C_ of” is present between the two marked entity mentions and negative if such a relation is not present. The resulting training set, after being balanced with respect to the mentions in each class, consists of 200 sentences, each one containing a different pair of marked entities. This collection of BERT models trained for different downstream tasks creates a rule-free pipeline for the automatic extraction of data from text.

### Supplementary information

Supplementary Information

## Data Availability

The test abstracts for the direct comparison with rule-based methods together with the datasets automatically extracted in the main and test extractions are available at: https://github.com/StefanoSanvitoGroup/BERT-PSIE-TC.

## Code availability

All the BERT-PSIE code necessary to run the automatic extraction of Curie temperatures and band gaps is available at https://github.com/StefanoSanvitoGroup/BERT-PSIE-TC.
