# Supplementary material for: A rule-free workflow for the automated generation of databases from scientific literature
Source: NPJ Comput Mater. 2023 Dec 13;9(1):222. doi: 10.1038/s41524-023-01171-9 (PMC11041762; doi:10.1038/s41524-023-01171-9)
Supplement: Supplementary file 1 — Supplementary Information [file 41524_2023_1171_MOESM1_ESM.pdf]

Supplementary Information: A rule-free workflow for the automated generation of databases from scientific literature

Luke P. J. Gilligan†,[1] Matteo Cobelli†,[1] Valentin Taufour,[2] and Stefano Sanvito[1,*]

[1]School of Physics, AMBER and CRANN Institute, Trinity College, Dublin 2, Ireland

[2]Department of Physics and Astronomy, University of California, Davis, California 95616, USA

I. SUPPLEMENTARY METHODS

Hyper-parameters have been optimized over the validation set, although we did not identify any particularly critical parameters that would significantly impact the final performance of the models. In other words the results are consistent over a range of parameters. For all models, we have used a training batch size of 32 and the learning rate used for the classifier, NER, and relation models are respectively $5 \cdot 10^{-5}$, $5 \cdot 10^{-5}$ and $2{\cdot}10^{-6}$. We have used a gradient clipping with a cut-off of 8 for all models. We have adopted early stopping with respect to the validation set loss as a strategy to prevent overfitting. The validation sets performance of the different models are reported in Table I and Table II.

II. SUPPLEMENTARY DISCUSSION

Additional comparison between BERT-PSIE and ChemDataExtractor, T_{C}.

In the main paper, we have compared our database of Curie temperatures, generated with BERT-PSIE, with a database generated with ChemDataExtractor [1]. However, the two extractions have been performed on different corpora. While an overlap is certainly present, since both works make use of the Elsevier API, this difference can impact the quantitative validity of the comparison. In order to eliminate such ambiguity, we have run ChemDataExtractor on the sentences deemed relevant by our classifier model. This comparison benchmarks the performance of our workflow, when we replace the NER model and the relation classifier with an extraction purely based on grammar rules, such as the one provided by ChemDataExtractor. We report in figures 1 and 2 the results of the query test and of the random-forest predictions, respectively. Both tests have been performed as described in the main text. Clearly the two automatically generated datasets appear to perform quite equally. Such a result reinforces the conclusion that BERT-PSIE has the ability to generate databases of similar quality as that of rule-based methods without the need to explicitly construct the grammar rules.

Additional comparison between BERT-PSIE and ChemDataExtractor, band gap.

In order to evaluate the automatic construction of the band-gap database, again we perform a comparison between BERT-PSIE, a dataset created with ChemDataExtractor and a manually curated one. The manually curated dataset is contained in the work of Zhuo *et al.* [2], where data from a range of sources [3–6] were manually aggregated. In contrast, a band-gap database created with a rules-based method is available in the work of Dong *et al.* [7]. This, however, contains data obtained from parsing natural language and from tables included in the targeted literature. Since, tables are not extracted with BERT-PSIE, a fair comparison is obtained by running the hybrid ChemDataExtractor model from reference [7] on the same corpus analysed by BERT-PSIE. In this way, BERT-PSIE and ChemDataExtractor work on an identical dataset of publications.

The comparison between the distribution of band-gap values and the relative abundance of elements within compounds of the databases can be appreciated in Fig. 3. The top panel demonstrates a striking similarity between the distributions of band gaps contained in the three datasets. However, we notice some bias in the manually extracted database towards compounds with band gaps of approximately 2.5 eV. This can be potentially explained by looking at the distribution of the relative elemental abundance in the bottom panel of the same figure. The manually curated dataset, in fact, features several dominant peaks that are not contained in the automatically extracted ones. These are likely associated to compounds contained in historical literature, but not in the most recent one searched by the CrossRef API. This fact further confirms the bias of curated databases towards selected sources within a given corpus and highlights the need to diversify the literature for general ML applications, for both the manually curated and the automated case. Finally, Fig. 4 reports results from the query test and from the RF model for the band-gap data extracted with BERT-PSIE (see main paper for details).

* Corresponding Author: sanvitos@tcd.ie

† Equal contribution.

III. SUPPLEMENTARY TABLES

Supplementary Table I. Validation set performance of the three modules developed for the T_C extraction: the sentence-level relevancy classifier, the NER and the relation classifier. Here we report: precision, P, recall, R, and F_1 score. The sizes of the validation sets (ValS) are also given (number of sentences used). For the case of NER, we report results for both chemical entities (Chem) and T_C, as well as the support.

Model	Entity	P	R	F_1	Support	ValS
Classifier		0.75	0.99	0.86		394
NER	Chem	0.91	0.95	0.93	1497	240
	T_C	0.74	0.92	0.82	603	240
Relation		0.81	0.66	0.73		100

Supplementary Table II. Validation set Performance of the three modules developed for the band gap extraction: the sentence-level relevancy classifier, the NER and the relation classifier. Here we report: precision, P, recall, R, and F_1 score. The sizes of the validation sets (ValS) are also given (number of sentences used). For the case of NER, we report results for both chemical entities (Chem) and Band Gap, as well as the support.

Model	Entity	P	R	F_1	Support	ValS
Classifier		0.96	1.00	0.98		134
NER	Chem	0.77	0.96	0.86	904	878
	Band Gap	0.78	0.87	0.82	145	
Relation		0.89	0.91	0.90		100

IV. SUPPLEMENTARY FIGURES

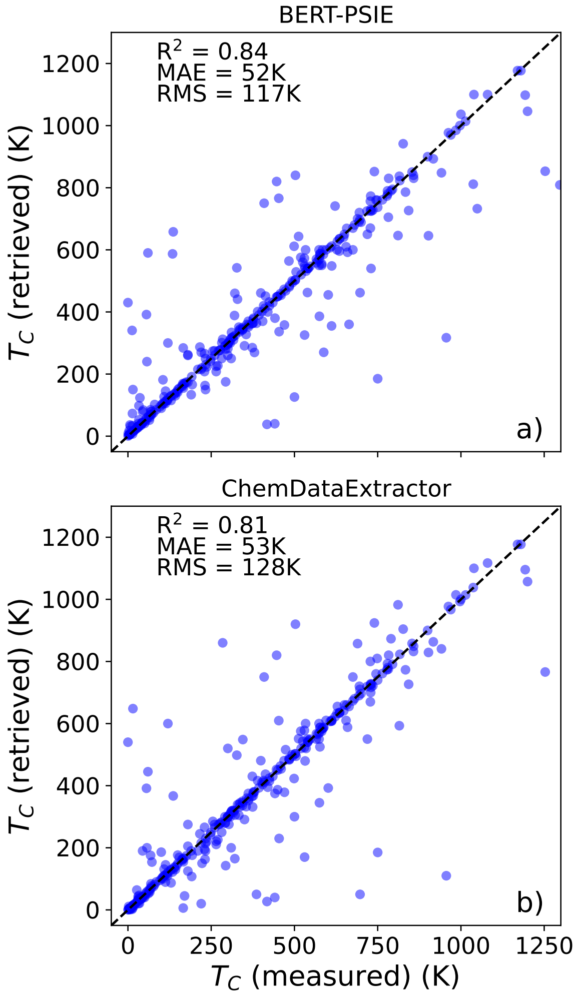

Supplementary Figure 1. Comparison between the T_C queried in the dataset automatically generated by BERT-PSIE and the values contained in the manually curated dataset **(a)**. The same comparison is performed on the dataset generated by running ChemDataExtractor on the same sentences deemed relevant by our BERT classifier **(b)**. The comparison is performed over the 322 compounds that are shared by both datasets.

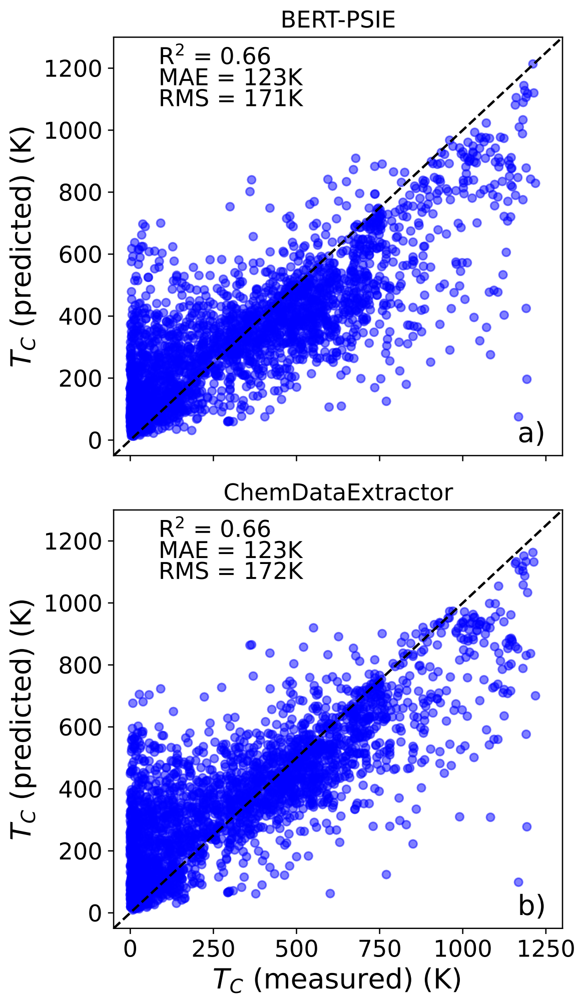

Supplementary Figure 2. Parity plot (predicted T_C vs manually extracted T_C) for the best RF compositional model constructed on the BERT-PSIE dataset **(a)** and on the dataset generated running ChemDataExtractor on the same sentences deemed relevant by our BERT classifier **(b)**. The test set consists of the 2,885 compounds that are not present in any of the two datasets, but for which we have a T_C manually extracted from the scientific literature.

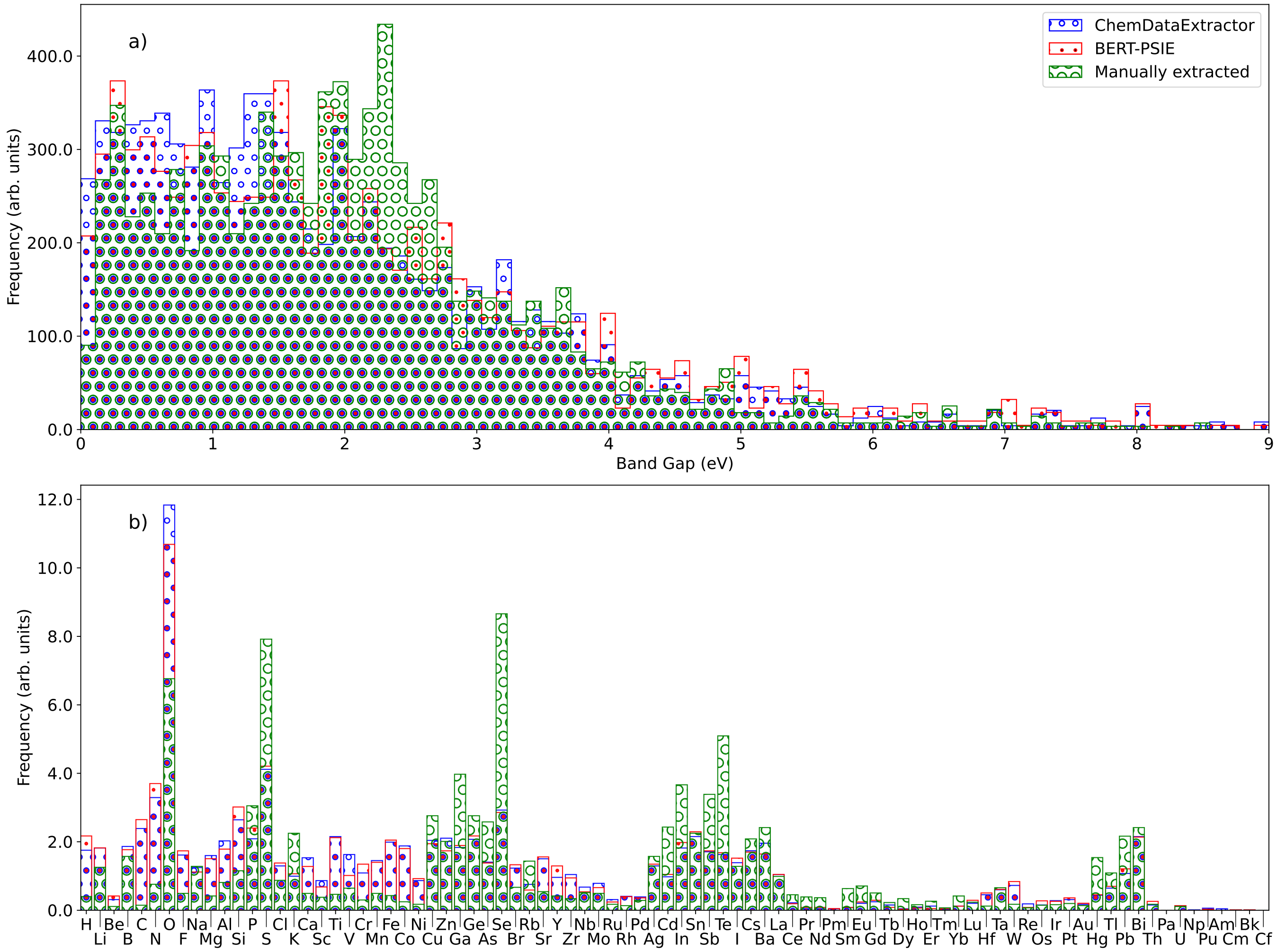

Supplementary Figure 3. Comparison between the content of the different band-gap databases: *(red box)* BERT-PSIE, *(blue box)* ChemDataExtractor and *(green box)* the manually-extracted database of Ref. [2]. **(a)** Normalized distribution of the band gaps extracted. **(b)** Relative elemental abundance across the compounds present in a database. Note that the automatically extracted datasets and the manually curated one are based on different literature libraries.

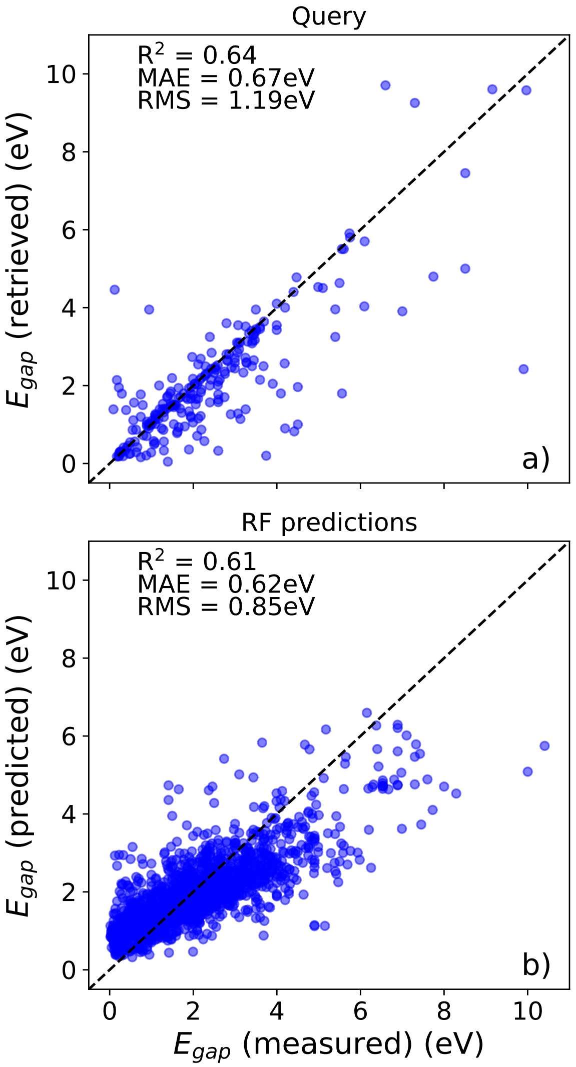

Supplementary Figure 4. Comparison between the band gaps queried from the dataset automatically generated by BERT-PSIE and the values contained in the manually curated dataset **(a)**. Parity plot for the best RF compositional model constructed on the BERT-PSIE dataset. The test set consists of the 2046 compounds that are not present in the dataset but for which we have a band gap from the manually curated corpus **(b)**.

[1] Court, C. J. & Cole, J. M. Auto-generated materials database of Curie and Néel temperatures via semi-supervised relationship extraction. *Sci. Data* **5**, 180111 (2018). URL https://doi.org/10.1038/sdata.2018.111.

[2] Zhuo, Y., Mansouri Tehrani, A. & Brgoch, J. Predicting the band gaps of inorganic solids by machine learning. *The Journal of Physical Chemistry Letters* **9**, 1668–1673 (2018). URL https://doi.org/10.1021/acs.jpclett.8b00124. PMID: 29532658, https://doi.org/10.1021/acs.jpclett.8b00124.

[3] Kiselyova, N. N., Dudarev, V. A. & Korzhuyev, M. A. Database on the bandgap of inorganic substances and materials. *Inorg. Mater. Appl. Res.* **7**, 34–39 (2016).

[4] Strehlow, W. & Cook, E. L. Compilation of energy band gaps in elemental and binary compound semiconductors and insulators. *J. Phys. Chem. Ref. Data* **2**, 163–200 (1973).

[5] Joshi, N. *Photoconductivity: art: science & technology*, vol. 25 (CRC Press, 1990).

[6] Madelung, O. *Semiconductors: data handbook* (Springer Science & Business Media, 2004).

[7] Dong, Q. & Cole, J. M. Auto-generated database of semiconductor band gaps using ChemDataExtractor. *Sci. Data* **9**, 193 (2022). URL https://doi.org/10.1038/s41597-022-01294-6.
